# Editorial: Pediatric Specificities of Medical Liability: Improvement Measures in Pediatric Settings

**DOI:** 10.3389/fped.2021.667020

**Published:** 2021-05-24

**Authors:** Paola Frati, Raffaele La Russa, Marzia Duse

**Affiliations:** ^1^Department of Anatomical, Histological, Forensic and Orthopaedic Sciences, Sapienza University of Rome, Rome, Italy; ^2^Istituto di Ricovero e Cura a Carattere Scientifico (IRCSS) Neuromed Mediterranean Neurological Institute, Pozzilli, Italy; ^3^Department of Clinical and Experimental Medicine, University of Foggia, Foggia, Italy

**Keywords:** medical liability, legal medicine, neurosurgery, health and safety, risk managemenent, autopsia

Medical liability is a major issue in the health care system, involving both legal and medical professions. Pediatric is at high risk of social blame in case of malpractice because the victims are children. The aim of the Frontiers in Pediatrics Research Topic about medical liability andimprovement measures in pediatric setting, was to perform an analysis of current issues about this theme and provide tools and solutions for medico-legal and clinical practice.

A problem that often arises in cases of alleged medical malpractice with a fatal outcome for children and infants, especially in civil trials, is that of not having performed an autopsy examination. Bertozzi et al. exhibited a new case series of autopsies on infants for which sudden death had arisen a dispute over emergency care. The response of the histopathological investigation had confirmed in all cases the diagnosis of acute bronchiolitis, exonerating doctors from any liability. Similarly, Di Nunno et al. have published an interesting case report on another case of alleged malpractice for the sudden death of a 4 year-old child. The autopsy made it possible to identify a compressive syndrome supported by a thymoma, a very rare pathology given the patient's age, as the cause of death. Also in this case the outcome of the autopsy led to the exclusion of medical liability. In both studies, the authors concluded for indispensability of medico-legal autopsy and histological examination in order to clarify the causal relationships between medical conduct and event.

By the way, litigation involving pediatrics is not only about fatal cases. Nonetheless, although the frequency of malpractice claims against pediatricians is among the lowest of all specialties, the mean malpractice indemnity payments paid by pediatricians are among the highest. This can happen also following limb fractures, which are common in pediatric orthopedics. Donati et al. investigated the case of poor out come in obese children; they discussed scientific literature highlighting possible reasons for inadequacy of current standard for treatment. The perspective coming from the paper argues in favor of guideline updates to improve healthcare quality and reduce litigation.

Furthermore, the scope of possible medical liability for pediatricians is not limited to the diagnosis and treatment of pathological pictures. This medical specialty may be involved in providing medical care to victims of abuse and violence so that a specific omission or underestimation can be grounds for reiteration of abuse. Turillazzi et al. presented the data collected through the application of the “pink code” at the local hospital, selecting cases involving minors. The “pink code” is a procedure implemented in the emergency departments that makes it possible to intercept circumstances of violence and abuse against weak subjects. The authors were able to see the effectiveness of this tool as long as physicians apply it carefully. The contrary case can lead to precise and serious professional liabilities.

Given the complexity of today's healthcare system, successful outcome depends on a range of factors. Patients' participation to care planning is one of these factors. Scopetti et al. explained that good communication among the pediatricians, the patients and their parents should not be left to personal sensitivity while it needs specific procedural tools as “shared decision making” and “advanced care planning.” This structured health-care relationship implemented through specific recommendations of good practice allows promotion of self-determination and sharing of therapeutic choices. A non-negligible aspect of the communication between doctor and patient in the field of pediatric care outside the hospital is that of the telephone conversation which can become a cause for claims. Blumberg et al. considered the development of these skills for their pediatric residents worthy of a targeted training intervention, notably in carrying out clinical triage over the phone. Jackson et al., instead, tried an innovative approach in the field of clinical trials, testing a blended research-design approach to co-design multimedia informed consent prototypes for experimental vaccine studies targeted at the pediatric population. They gained interesting insights using the participatory approach called “design thinking” and combining data obtained from social media analysis, a survey and workshops. This approach is clearly helpful to provide clearer information and to better address to children and families.

An unsolved problem in biolaw is that of so called “biobanks.” Cannovo et al. discuss about uncertainties in definitions and legal status for these non-profit organizations, especially both for what concerns databases about clinical and personal information, and what concerns biological samples. The authors focus on the situation in Italy and propose that biological samples be considered as supra-individual goods at the service of the community. The implications of biobanks concerning genetic material and procreation have inevitable repercussions in the field of pediatrics and related medical liability.

The collection of articles on medical liability in pediatrics in this Frontiers Research Topic highlight our current understanding about risk management techniques applicable to pediatric specificities. Key areas emerging as future research priorities include adapting clinical guidelines to pediatric population. There is a major need to improve communication strategies to address to parents and children sharing therapeutic choices, but also to provide adequate informed consent for experimental trials. Furthermore, it is of Paramount importance to complete resident training in every aspect of clinical practice, including telephone communication. Finally, both society and health-service should recognize importance of performing full autopsies in fatal cases involving pediatrics care in order to assess causes of death and relationship between medical conduct and events.

## Author Contributions

PF and MD contributed to conception and design the editorial. RL wrote the first draft of the manuscript. All authors contributed to the article and approved the submitted version.

## Conflict of Interest

The authors declare that the research was conducted in the absence of any commercial or financial relationships that could be construed as a potential conflict of interest.

